# A tail of translational regulation

**DOI:** 10.7554/eLife.29104

**Published:** 2017-06-27

**Authors:** Gillian A Gray, Nicola K Gray

**Affiliations:** 1 BHF/University Centre for Cardiovascular Science, Queen's Medical Research Institute, University of Edinburgh, Edinburgh, United Kingdom; 2 MRC Centre for Reproductive Health, Queen's Medical Research Institute, University of Edinburgh, Edinburgh, United KingdomNicola.Gray@ed.ac.uk

**Keywords:** post-transcriptional gene regulation, translation control, polyadenylation, cardiac hypertrophy, postnatal heart development, protein synthesis, Mouse

## Abstract

An RNA-binding protein called PABPC1 has an important role in determining protein synthesis rates and hypertrophy in the heart.

**Related research article** Chorghade S, Seimetz J, Emmons R, Yang J, Bresson SM, De Lisio M, Parise G, Conrad NK, Kalsotra A. 2017. Poly(A) tail length regulates PABPC1 expression to tune translation in the heart. *eLife* **6**:e24139. doi: 10.7554/eLife.24139

The mammalian heart is a muscular pump that handles about five litres of blood per minute in the average human. Muscle cells known as cardiomyocytes make up 25–35% of all cells in the heart, with the remainder largely being vascular endothelial cells and fibroblasts (Pinto et al., 2016). The proliferation of cardiomyocytes is key to the growth of the heart before birth, and also soon after birth, but most cardiomyocytes lose their capacity to proliferate after the early post-natal period in both mice (Soonpaa et al., 1996) and humans (Bergmann et al., 2015). Thereafter, cardiac growth is achieved mainly by increases in the size of cardiomyocytes in a process known as hypertrophy.

In adulthood cardiac hypertrophy can occur as a physiological adaptation to an increased need to pump blood following sustained athletic training or during pregnancy (reviewed in Maillet et al., 2013). Cardiac hypertrophy can also be pathological in nature – for example, when it is caused by long-term hypertension. Although cellular signalling pathways and gene transcription change in distinct ways during 'physiological' and 'pathological' cardiac hypertrophy, both require *de novo* protein synthesis in order to increase cardiomyocyte size (Figure 1; reviewed in Heineke and Molkentin, 2006). Now, in eLife, Auinash Kalsotra of the University of Illinois and co-workers – including Sandip Chorghade and Joseph Seimetz as joint first authors – reveal an unexpected role for an RNA-binding protein called PABPC1 in cardiac hypertrophy (Chorghade et al., 2017).

**Figure 1.**
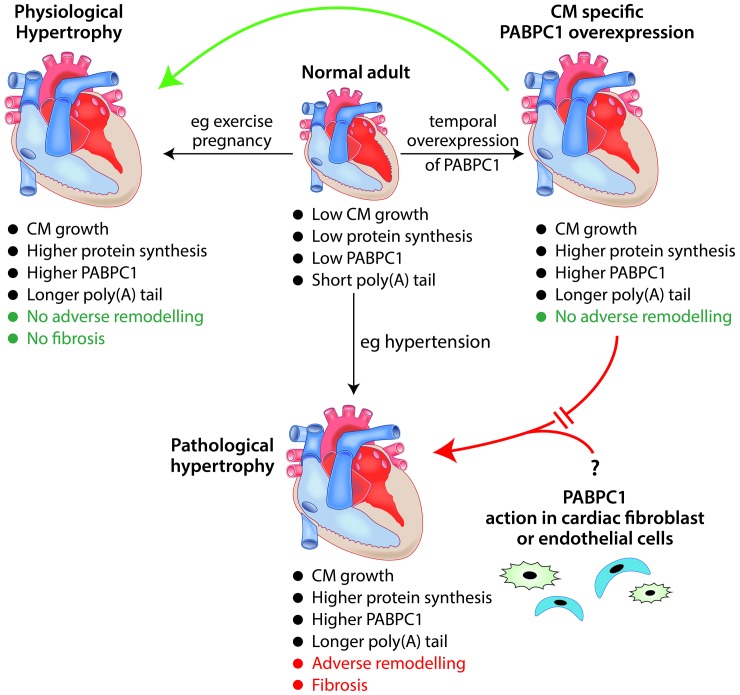
The role of the RNA-binding protein PABPC1 in cardiac hypertrophy. In a normal adult heart, cardiomyocytes (CM) show little growth or turnover, and protein synthesis and PABPC1 levels are low. The temporal overexpression of PABPC1 in adult cardiomyocytes leads to a form of cardiac hypertrophy (top right) that resembles (green arrow) physiological cardiac hypertrophy (e.g. induced by pregnancy or endurance exercise) but not (red broken arrow) pathological cardiac hypertrophy (e.g. induced by long-term hypertension). However, a number of features (such as cardiomyocyte growth, higher protein synthesis and higher levels of PABPC1) are common to both physiological and pathological cardiac hypertrophy. This raises the question (bottom right) of whether PABPC1 function in other types of cardiac cells (such as endothelial cells or fibroblasts) may be important in the induction of pathological cardiac hypertrophy. During cardiac hypertrophy, longer poly(A) tails are associated with more efficient translation of the messenger RNA for PABPC1.

Illustration: Ronnie Grant

PABPC1 (commonly known as PABP1) is one of the 1000+ RNA-binding proteins found in humans, and has multiple functions in controlling the synthesis of new proteins (reviewed in Smith et al., 2014). However, it is best characterized as a factor that is required for efficient messenger RNA (mRNA) translation and to maintain mRNA stability, and it is generally considered to be an abundant and essential protein (Smith et al., 2014).

Protein synthesis is surprisingly low in the adult heart, perhaps reflecting the relatively low level of cell turnover there. Intriguingly, Chorghade et al. show that PABPC1 is abundant in the neonatal heart, but is only present at very low levels in the adult heart, and they go on to show that the low levels of PABPC1 in the adult heart are due to reduced translation of PABPC1 mRNA. Chorghade et al. also found that this reduction in translation coincided with a shortening of the 3’ poly(A) tail of PABPC1 mRNA: poly(A) tails are critical for efficient translation but their length can be exquisitely regulated by a number of mechanisms (reviewed in Charlesworth et al., 2013).

Intriguingly, the length of the poly(A) tail of PABPC1 mRNA and its translation were increased in experimental models of both physiological hypertrophy (induced by swimming) and pathological hypertrophy (induced by narrowing of the aorta to mimic high blood pressure), leading to an increase in PABPC1 levels.

It was already known that the translation of PABPC1 mRNA was controlled by two mechanisms (reviewed in Eliseeva et al., 2013), but uncovering that its poly(A) tail length is also regulated further underscores how important it is to control cellular levels of PABPC1. However, Chorghade et al. found that the 3’untranslated region of PABPC1 mRNA is not important for its regulated translation. This is surprising since studies in multiple cell types have shown that sequences within the 3’untranslated region normally regulate poly(A) tail length. Therefore, more work is needed to clarify the mechanism by which PABPC1 mRNA translation and poly(A) tail length is controlled in cardiomyocytes.

The altered levels of PABPC1 suggested that it may facilitate a dynamic response to an increased workload being placed on the heart. Importantly, Chorghade *et al*. probed the functional relevance of altered PABPC1 levels and in doing so uncovered new avenues for exploration. Reducing PABPC1 levels in cultured neonatal mouse cardiomyocytes blocked the normal increase in protein synthesis and cell size associated with stimulus-induced growth: moreover, this effect that could be rescued with wild-type PABPC1, but not with mutant PABPC1.

In transgenic mice, the overexpression of PABPC1 in adult cardiomyocytes increased protein synthesis and heart size without any loss of cardiac function, similar to what happens in physiological hypertrophy (Figure 1). The transcriptional changes that accompanied these increases were also similar to physiological rather than pathological hypertrophy. Therefore, despite the level of PABPC1 increasing in models of both forms of hypertrophy, increased PABPC1 levels in cardiomyocytes do not appear sufficient to drive pathological hypertrophy.

Other types of cardiac cells, including endothelial cells and fibroblasts, proliferate during pathological hypertrophy and can influence the behaviour of cardiomyocytes (Figure 1; Frieler and Mortensen, 2015). Future research is needed to determine the extent to which PABPC1 levels in other cardiac cell types may be involved in this process.

In addition to adding to the growing body of evidence that translational control has a role in cardiac hypertrophy, the work of Chorghade et al. also greatly extends our knowledge of the physiological roles of PABPC1. Previous whole-organism studies of PABPC1 function in vertebrates were confined to the frog *X. laevis*, where it is essential for early embryonic development (Gorgoni et al., 2011).

Since these new results also showed that PABPC1 levels were highest in fetal and neonatal hearts (prior to hypertrophic growth), this suggests that PABPC1 might have a critical role in cell proliferation and/or the growth of heart tissue during heart development and maturation, as well as in adult cardiac hypertrophy. Thus, ascertaining its function in fetal and neonatal hearts in mammals is an exciting question for the future.

Finally, there is significant interest in invoking pathways that promote physiological cardiac hypertrophy as an alternative to the detrimental structural and functional “remodelling” of the heart that is associated with heart failure. The work of Chorghade et al. has opened a new window on our understanding of cardiac hypertrophy, and future research should reveal whether or not PABPC1 has potential as a therapeutic target for cardiovascular disease.
